# Measuring the Budget Impact of Nondiscriminatory Cost-Effectiveness

**DOI:** 10.1001/jamahealthforum.2025.3076

**Published:** 2025-09-05

**Authors:** Karen Mulligan, Drishti Baid, Maria-Alice Manetas, Darius N. Lakdawalla

**Affiliations:** 1 Sol Price School of Public Policy, University of Southern California, Los Angeles; 2 Leonard D. Schaeffer Center for Health Policy and Economics, University of Southern California, Los Angeles, California; 3 Alfred E. Mann School of Pharmacy and Pharmaceutical Sciences, University of Southern California, Los Angeles, California

## Abstract

**Importance:**

The US Inflation and Reduction Act (IRA) prohibits the Centers for Medicare & Medicaid Services (CMS) from using discriminatory methods such as cost-effectiveness analysis (CEA) that assign lower value to treating sicker and disabled persons. Generalized risk–adjusted cost- effectiveness (GRACE) provides a nondiscriminatory alternative, but the potential impact on health care budgets is unknown.

**Objective:**

To compare value-based drug prices based on traditional CEA with those based on IRA-compliant GRACE and assess the implications for health care budgets.

**Design and Setting:**

In this economic evaluation, GRACE was implemented using the direct-utility method and estimated the resulting value-based prices and total budget impact. Model inputs were derived from CEAs published by the Institute for Clinical and Economic Review (ICER) between 2014 and 2024. Data from 302 CEA results for pharmaceuticals published across 72 studies were extracted. The final analysis sample consisted of 259 observations (219 treatment-comparator pairs) across 53 distinct diseases, some of which had subgroup results.

**Main Outcomes and Measures:**

Value-based prices under GRACE and CEA were estimated. A 1-year budget impact was calculated, measured as total drug expenditures using value-based prices assuming a willingness-to-pay threshold of $150 000. The data were analyzed from October 2024 to May 2025.

**Results:**

The mean value-based prices were 7.5% higher under GRACE than under CEA (IQR, −3.9% to 9.1%). Furthermore, compared with traditional CEA, GRACE increased value-based prices for more severe diseases and decreased them for milder diseases. Twenty-four drugs (8 from the top population size quartile) cost less under GRACE; total spending was 3.3% lower under GRACE for these drugs. The remaining 45 drugs (13 from the bottom population size quartile) cost more under GRACE, resulting in 14.7% higher spending for these drugs. Taken together, GRACE increased the total budget by 2%..

**Conclusions and Relevance:**

This economic evaluation found that although GRACE does increase value-based prices on average, the net effect on total health care spent is minimal, in part because resources are redistributed toward more severe, less prevalent illnesses.

## Key Points

**Question:**

What is the net effect on health care budgets of relying on a nondiscriminatory generalized risk-adjusted cost-effectiveness (GRACE) framework to establish value-based prices rather than traditional cost-effectiveness analysis (CEA)?

**Findings:**

This economic evaluation found that GRACE increases value-based prices for more severe diseases but decreases them for less severe ones. On net, using GRACE value-based prices is approximately budget-neutral compared with the use of CEA value-based prices.

**Meaning:**

GRACE provides an alternative to CEA that complies with US federal prohibitions on discriminatory cost-effectiveness methods, facilitating transparency in price-setting for drugs without sacrificing the overall cost-efficiency gains of traditional CEA.

## Teaser

This economic evaluation assesses value-based prices by comparing cost-effectiveness analysis with generalized risk–adjusted cost- effectiveness for treating patients.

## Introduction

Health economists use traditional cost-effectiveness analysis (CEA) with quality-adjusted life-years (QALYs) to identify resource-efficient health interventions. However, the US Inflation and Reduction Act (IRA) stipulates that Centers for Medicare & Medicaid Services (CMS) “shall not use evidence from comparative clinical effectiveness research in a manner that treats extending the life of an elderly, disabled, or terminally ill individual as of lower value than extending the life of an individual who is younger, non-disabled, or not terminally ill.”^1^

Generalized risk–adjusted cost-effectiveness (GRACE) provides a nondiscriminatory alternative to CEA grounded in economics. Importantly, it allows the value of health gains to vary with health (eg, gains are more valuable to people with more severe illnesses) and accounts for the way baseline disability increases the value of health gains.^2^ GRACE can be implemented in a manner that assigns the same value of life-extension to disabled and nondisabled persons, which allows CMS to use it under the IRA.^3,4^

Compared with CEA, GRACE lowers value-based prices for mild conditions but increases them for severe ones,^5^ but its net budget impact is unknown. To illuminate this issue, aggregate spending implied by GRACE and by CEA were compared, which was used as a budgetary benchmark, given its established role among international payers and health technology assessment bodies. By analyzing drugs previously evaluated by the Institute for Clinical and Economic Review (ICER), a nondiscriminatory GRACE framework comparing value-based prices under both approaches to quantify its incremental budget impact was implemented.

## Methods

Decision-makers use CEA to determine whether a new treatment’s benefits over the current standard of care justifies its cost. CEA measures benefit in some unit of health gain, typically QALYs. One year of perfect health produces one QALY, and illness scales this value down. CEA calculates what a new treatment costs per additional QALY produced and compares this to an assumed value of a QALY. Suppose drug A is the current treatment for a disease, drug B is introduced, and a QALY is assumed to be worth $150 000. Drug B is cost-effective if $150 000 > (cost_B_ − cost_A_) / (QALY_B_ − QALY_A_).

QALYs presume that life extension is worth less to sicker people, raising concerns about discrimination against older or disabled individuals. CEA also presumes that baseline health does not influence QALY value, so an additional QALY is worth the same to a person in good or poor health. GRACE allows baseline health to influence value, letting preference data dictate whether health is worth more to people with less of it. GRACE also recognizes that healthier people may be less willing to spend on health improvement. These 2 generalizations modify the decision rule: $150 000 × (disability adjustment factor) > (cost_B_ − cost_A_) / (U(health_B_) − U(health_A_).

The disability adjustment factor is 1 for perfectly healthy populations and rises with preexisting disability. *U* represents what economists call a *utility function*. CEA is the special case of GRACE with linear utility, under which health benefits are worth the same regardless of baseline health. A more detailed derivation appears in Lakdawalla and Phelps.^6^

More details appear in the eMethods section of Supplement 1. This study was conducted in accordance with the Consolidated Health Economic Evaluation Reporting Standards (CHEERS) reporting guidelines. This study did not require institution review board approval because it did not involve human subjects research. We extracted data for 165 intervention drugs (249 intervention-comparator combinations) across 57 distinct diseases from 72 ICER reports that evaluated pharmaceuticals and were published between 2014 and 2024.^7^ We extracted 4 outcomes for both the intervention and comparator arms: total life-years, total QALYs, total cost, and total drug cost. We also extracted the assumed discount rate and currency base year, and the number of treatment-eligible patients ICER used to measure budget impact. The data were analyzed from October 2024 to May 2025.

The value-based price is the price of the intervention drug where its incremental costs equal incremental benefits. We compared GRACE and CEA value-based prices at the drug-comparator-disease-subgroup level for 259 observations (219 treatment-comparator pairs) across 53 diseases. If preferences align with the “power” utility function (ie, *U*(*h*) = *h^α^*), theory implies GRACE produces nondiscriminatory outcomes, because the value of life extension does not vary with baseline health.^4^ Therefore, we implemented GRACE assuming power utility and using the “exact utility” approach,^6^ using published utility parameters estimated from a representative US population.^5^ GRACE calculations required a disability adjustment and 4 components for the intervention and comparator: survival probabilities, total discounted costs, and utility. In cases where ICER reports do not publish a relevant parameter, we rely on assumptions to approximate it, including constant mortality hazards and health levels. Supplement 1 describes the GRACE implementation and approximation methods in detail.

To validate our approximating assumptions, we replicated ICER’s results under traditional CEA. We also assessed sensitivity to the assumption of constant health. We calculated the total value, risk-adjusted willingness to pay threshold, and GRACE value-based prices, assuming that a QALY is worth $150 000. Scenario analyses were conducted using QALY values of $50 000, $100 000, and $200 000.^8^

The 69 observations with both population size and value-based price data were included in the budget impact analysis. We calculated total expenditures for each drug-disease combination as the total annual treatment-eligible population multiplied by value-based prices. We stratified results by disease severity, measured as average health in the comparator (ie, standard of care) arm.

## Results

The mean value-based price is 7.5% higher under GRACE than CEA (IQR, −3.9% to 9.1%). The Figure presents the percentage difference between GRACE and CEA value-based prices by disease severity, for all observations and only those with nonmissing treatment-eligible populations. As expected, GRACE increases value-based prices for more severe diseases and decreases value-based prices for less severe diseases., compared to traditional CEA.

**Figure.**
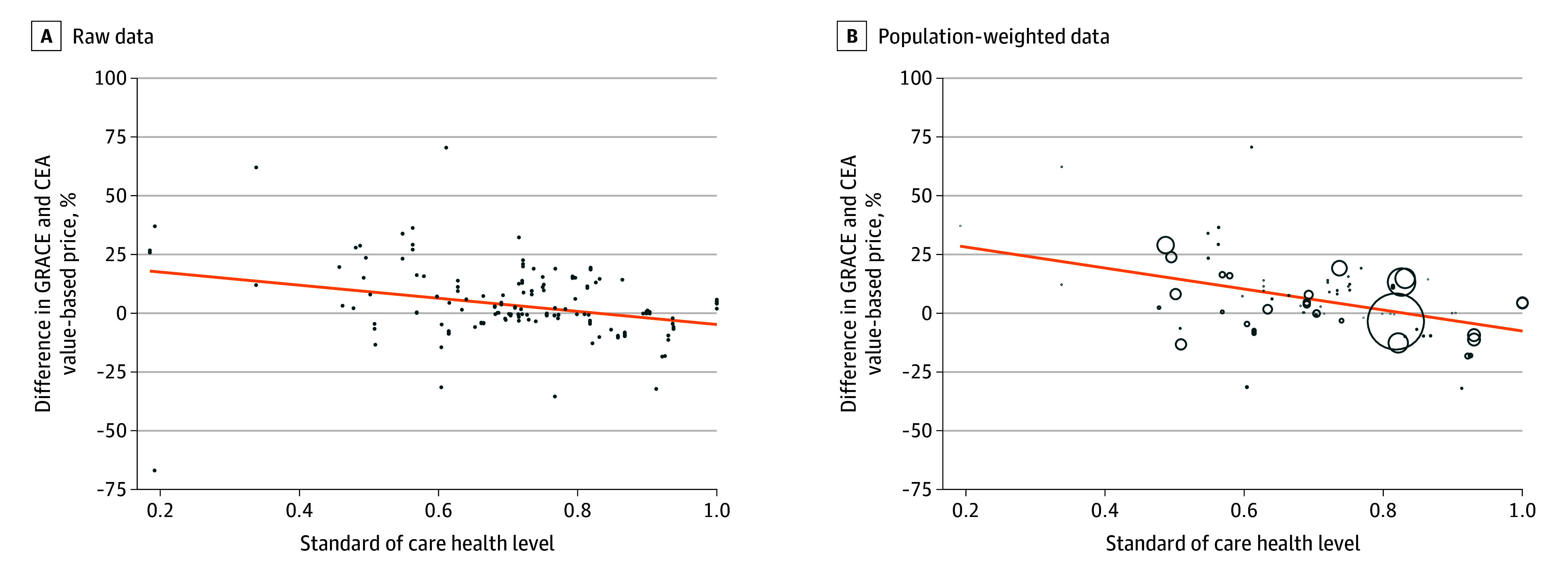
Difference in Generalized Risk-Adjusted Cost-Effectiveness (GRACE) and Cost-Effectiveness Analysis (CEA) Value-Based Prices A, All observations with value-based prices (N = 186). B, All observations with value-based prices and information on patient population size (N = 84). The size of the circles indicates the size of the treated population.

In the Table, we present the budget impact analysis for 69 drugs with nonmissing population data, assuming QALYs are worth $150 000. Twenty-four drugs (8 from the top population size quartile) cost less under GRACE; total spending is 3.3% lower under GRACE for these drugs. The remaining 45 drugs (13 from the bottom population size quartile) cost more under GRACE, resulting in 14.7% higher spending for these drugs. Taken together, GRACE increases total spending by 2%, consisting of 16.6% higher spending on cost-effective drugs and 2.5% lower spending on cost-ineffective ones. GRACE also shifts resources from milder illness toward more severe illness. If we assume QALYs are worth $100 000 or $200 000, GRACE instead increases total spending by 1.8% or 2.2%, respectively. Results were similar when relaxing the constant health assumption. Additional scenario analyses are reported in the eAppendix in Supplement 1.

**Table.** Spending Distribution by Disease Severity^a^

Standard of care health level (0-100)	Cost-effectiveness analysis, %			Generalized risk-adjusted cost-effectiveness, %		
	All drugs (n = 69)	Cost-ineffective drugs (n = 44)	Cost-effective drugs (n = 25)	All drugs (n = 69)	Cost-ineffective drugs (n = 42)	Cost-effective drugs (n = 27)
<60 (n = 12)	3.78	1.07	12.71	4.41	1.28	13.03
60-69 (n = 19)	2.25	1.72	4.01	2.24	1.99	2.93
70-79 (n = 17)	6.05	7.13	2.48	6.88	8.46	2.54
80-100 (n = 21)	87.92	90.08	80.80	86.47	88.27	81.51
Total spend (billions), $	444.5	340.9	103.6	453.4	332.7	120.7

^a^ Analysis includes drugs with nonmissing data for population size and value-based prices. We assume the drug price is equal to the value-based prices and 100% of the eligible population receives treatment.

## Discussion

The IRA prohibits traditional CEA, yet the lack of a systematic and transparent price-determination process muddies incentives for innovators and other stakeholders. The results of this analysis suggest that systematic, nondiscriminatory value assessment produces budgetary implications similar to traditional CEA, despite higher value-based prices for drugs treating more severe illness.

We compare GRACE with CEA-based pricing, which itself saves costs over the status quo.^9^ Thus, even though its 2% cost increase translates to meaningful absolute dollars, GRACE likely saves considerable resources over the status quo. Moreover, adopting GRACE has important implications for innovation incentives. If manufacturers expect broad use of GRACE, they may shift development away from less severe to more severe illnesses because value-based prices tend to be higher for severe illnesses.

### Limitations

The reliance on published ICER reports allowed for implementation of GRACE for a large sample of drugs simultaneously, but this has key limitations. Because we lacked access to underlying models, we relied on simplifying assumptions of constant mortality and uniformly distributed QALYs. Nevertheless, ICER’s results were replicated using approximation methods, lending credence to the approach. In addition, ICER tends to evaluate newer drugs that are relatively high cost. Future work should examine the value-based prices and budgetary implications of switching from CEA to GRACE in a representative sample of drugs.

## Conclusions

Facing limited health care budgets, payers—including CMS—must determine which drugs to cover and what prices to pay. Under the IRA, CMS is required to consider several factors (eg, research and development costs, sales volumes, relative drug effectiveness) in setting maximum fair prices.^10^ The degree to which therapeutic benefit is considered relative to other factors is unknown. Incorporating GRACE into price determinations would increase transparency in negotiations and better align pricing with value, providing more efficient incentives to innovators.

## Notes

Supplement 1.
eAppendix

Supplement 2.
Data Sharing Statement
