# Supplementary material for: Measuring the Budget Impact of Nondiscriminatory Cost-Effectiveness
Source: JAMA Health Forum. 2025 Sep 5;6(9):e253076. doi: 10.1001/jamahealthforum.2025.3076 (PMC12413641; doi:10.1001/jamahealthforum.2025.3076)
Supplement: Supplement 1. — eAppendix [file jamahealthforum-e253076-s001.pdf]

Supplemental Online Content

Mulligan K, Baid D, Manetas M-A, Lakdawalla DN. Measuring the budget impact of nondiscriminatory cost-effectiveness. *JAMA Health Forum*. Published online September 5, 2025. doi:10.1001/jamahealthforum.2025.3076

eAppendix.

This supplemental material has been provided by the authors to give readers additional information about their work.

© 2025 Mulligan K, et al. *JAMA Health Forum*.

eAppendix

This Appendix contains a more detailed description of our methods to supplement the manuscript content. Section 1 provides a brief overview of the data construction and GRACE methods, including key assumptions. Section 2 presents the relevant formulae from the GRACE theoretical framework and outlines the conceptual framework for implementation. Section 3 provides the analogous framework for the standard CEA implementation. Section 4 elaborates on the empirical implementation, including data requirements. We present model inputs and GRACE results for an example published CEA report in Section 5. Finally, results from our scenario analysis and robustness checks are presented in Section 6.

1. Methods overview

We extracted data from 302 cost-effectiveness analysis (CEA) results in 72 Institute for Clinical and Economic Review (ICER) reports that evaluated pharmaceuticals published between 2014 and 2024. We excluded evaluations that (1) were canceled or had yet to publish a final report at the time of our extraction, (2) reviewed non-pharmaceutical interventions, or (3) did not report outcomes in terms of both quality adjusted life years (QALYs) and life years (LYs). The final extracted data contained 165 intervention drugs (249 intervention-comparator combinations) across 57 diseases, some of which included subgroup results. We excluded 43 observations from our analysis due to missing data for LYs, QALYs, or costs for either the intervention or comparator drug.

We implement generalized risk-adjusted cost-effectiveness (GRACE) using the exact utility framework proposed in Lakdawalla and Phelps (2023).[1] GRACE calculations require five components: disability adjustment, survival probabilities for the intervention and comparator, total discounted costs for the intervention and comparator, and the utility associated with the intervention and the comparator. In cases where ICER reports do not publish a relevant parameter, we rely on assumptions to approximate it.

Following ICER, we assume the age- and gender-adjusted quality of life in the healthy population is 0.851; this corresponds to a GRACE pre-existing disability adjustment of 0.149, which translates to a 12.3% higher willingness to pay for health improvement.[2] Since survival probabilities are not typically reported, we approximate them using the total undiscounted LYs reported by ICER and assuming a constant mortality survival function; if undiscounted total LYs were not reported, we relied on discounted total LYs and adjusted them with the reported discount rate. Total discounted costs were extracted directly from the ICER reports. Since ICER does not typically report quality of life (henceforth referred to as health) in each period, we assumed uniform, average health, calculated as total discounted QALYs (reported by ICER) divided by total discounted LYs (constructed from survival probabilities and the discount rate) for the treatment and comparator arms. We evaluated benefits from treatment using a power utility function parameterized with estimates from Mulligan et al (2024).[3] A more detailed description of the GRACE implementation, including formulas and model inputs is provided in the next section.

2. GRACE Implementation Framework

For the remainder of the appendix, let j index each time period. Let the superscripts T and U indicate the intervention and comparator arm, respectively; more generally, we will index arms using $i=T,U$.

Our budget impact analysis relies on value-based prices (VBP) calculated under CEA and GRACE. The VBP is defined as the drug price that implies zero net monetary benefit (NMB, or total incremental benefits minus total incremental costs) for a given willingness to pay (WTP) threshold. In a multi-period model with an infinite time horizon comprising discrete periods of time indexed from $j = 0$ to $j = \infty$, NMB_{GRACE} can be expressed mathematically as follows:

© 2025 Mulligan K, et al. *JAMA Health Forum*.

Equation 2.1 GRACE NMB [1]

$$NMB_{GRACE} = \phi \sum_{j=0}^{\infty} \beta^j \left\{ \mu_{pj} \left[K \frac{W(H_{sj} + B_j)}{W(H_0(1 - d^*))} H_0 - Cost_j^T \right] + p_j^U \left[K \frac{W(H_{sj} + B_j) - W(H_{sj})}{W(H_0(1 - d^*))} H_0 - \Delta Cost_j \right] \right\}$$

Definitions of the individual terms in this expression appear in Table 1 below. Alternatively, the technology adoption decision rule can be expressed in terms of the incremental GRACE ratio as follows:

Equation 2.2 GRACE Decision Rule [2]

$$K \frac{H_0}{W(H_0(1 - d^*))} > \frac{\sum_{j=0}^{\infty} \beta^j [\mu_{pj} Cost_j^T + p_j^U \Delta Cost_j]}{\sum_{j=0}^{\infty} \beta^j \{[\mu_{pj} E[W(H_{sj} + B_j)] + p_j^U E[W(H_{sj} + B_j)] - E[W(H_{sj})]]\}}$$

where the left-hand side is the GRACE willingness to pay threshold for a unit of GRACE utility (GRACE WTP), and the right-hand side is the generalized risk- and severity-adjusted incremental cost-effectiveness ratio, measuring incremental costs per unit of incremental GRACE utility. Note that the numerator on the right-hand side of the expression is equivalent to total discounted incremental cost (i.e., intervention arm cost net comparator arm cost). This value is the same for CEA and GRACE. The denominator represents generalized risk- and severity-adjusted QALYs, which simplify to traditional QALYs in the special case where the utility function, W, satisfies $W(H) = H$. Each mathematical term in equations 2.1 and 2.2 is defined in Table 1. We point readers to Lakdawalla and Phelps (2023) for a full exposition of the exact utility formulation for implementing GRACE.[1]

Table 1. Definition of GRACE Parameters

Parameter	Description
K	WTP threshold from CEA
H_0	Baseline health (usually assumed to equal 1)
ϕ	Probability of illness occurring in period 1
H_{sj}	Health in the sick state in period j (corresponds to comparator arm)
B_j	Treatment benefit from intervention drug in period j
$H_{sj} + B_j$	Health in the treated state in period j (corresponds to the intervention arm)
d^*	Health loss from baseline disability
β	Single-period discount factor
p_j^T	Cumulative probability of surviving from period zero to period j for patients in the intervention group
p_j^U	Cumulative probability of surviving from period zero to period j for the comparator arm
μ_{pj}	Incremental probability of surviving from period zero to period j for the intervention arm ($\mu_{pj} \equiv p_j^T - p_j^U$)
$Cost_j^T$	Total cost for the intervention group in period j
$\Delta Cost_j$	Incremental cost (intervention – comparator) in period j
$W(H)$	Exact utility function over health

2.1 Assumptions and Simplified GRACE Equations

In keeping with ICER's typical approach, we assume an ex-post perspective, which implies the probability of illness occurring in period 1 is equal to 1 (i.e., $\phi = 1$); in any event, this is without loss of generality since ϕ does not affect VBPs. Furthermore, we assume perfect health at baseline (i.e., $H_0 = 1$).

[1] Refer to equation 22a in Lakdawalla and Phelps [1].

[2] Refer to equation 22c in Lakdawalla and Phelps [1].

Because we did not build models de novo, we apply several simplifying assumptions that allow us to implement the GRACE NMB and decision rule calculations using CEA results from published reports by ICER.

1. Health benefits are non-stochastic such that $E(H) = H$ and $E(W(H)) = W(H)$. We cannot implement the stochastic version of GRACE because we do not know the distribution of treatment benefits in ICER's underlying models.
2. One-period cost and health measures are constant across all periods, such that $Cost_0^i = Cost_1^i = \cdots = Cost_\infty^i \equiv Cost^i$ for each arm (*i* equals *T, U), $H_{s0} = H_{s1} = \cdots = H_{s\infty} \equiv H_s$, and $B_0 = B_1 = \cdots = B_\infty \equiv B$. We conducted sensitivity analysis around the assumption of constant health (results in Section 6).
3. We treat time as a continuous measure such that aggregate health gains and aggregate costs can be evaluated using an indefinite integral over an infinite time horizon from period $j = 0$ to $j = \infty$.[3]
4. We assume exponential discounting of health gains and costs over time with a fixed discount rate (r) per period. Thus, the effective discount factor in period j is $\beta_j = \exp(-rj)$.
5. We assume an exponential survival function of the form $S(j) = \exp(-\lambda j)$ where the parameter λ represents a constant annual mortality hazard. Mean survival is equal to $\frac{1}{\lambda}$, and the corresponding one-period survival probability between periods j and *(j+1)* is then given by $\pi = \exp(-\lambda)$. We can rewrite the cumulative survival probabilities for each arm as follows: $p_j^i = (\pi^i)^j = \exp(-\lambda^i j)$ for *i* equal to *T, U.*
6. We assume mean survival is equivalent to the total undiscounted life years (LY) for each arm, such that $\left(\frac{1}{\lambda^i}\right) = (total\ undiscounted\ LY)^i$ for *i* equal to T, U. Under Assumption #4, the mean discounted survival is given by $\frac{1}{\lambda^i + r}$, where *r* is the discount rate. We assume mean survival with discounting is equivalent to the total discounted LYs for each arm, such that $\frac{1}{\lambda^i + r} = (total\ discounted\ LY)^i$ for *i* equal to T, U.[4]

These assumptions imply that the NMB expression from equation 2.1 can be re-written as

$$NMB_{GRACE} = K\frac{W(H_s + B)}{W(1-d^*)}\int_{j=0}^{\infty}\exp(-rj)\left[[(\pi^T)^j - (\pi^U)^j]\right] - Cost^T\int_{j=0}^{\infty}\exp(-rj)\,[(\pi^T)^j - (\pi^U)^j]$$

$$+\ K\left[\frac{W(H_s + B) - W(H_s)}{W(1-d^*)}\right]\int_{j=0}^{\infty}\exp(-rj)\,(\pi^U)^j - [Cost^T - Cost^U]\int_{j=0}^{\infty}\exp(-rj)\,(\pi^U)^j$$

We note that $\int_{j=0}^{\infty}\exp(-rj)\,(\pi^i)^j) = \exp(-rj)\exp(-\lambda^i j) = \frac{1}{\lambda^i + r}$ for *i* equal to *T, U*, which under assumption #5 is equal to total discounted LYs for arm *i.* Collecting the cost and benefit (i.e. utility) terms and simplifying, we have:

$$NMB_{GRACE} = \frac{K}{W(1-d^*)}[W(H_s + B)\cdot total\ discounted\ LY^T - W(H_s)\cdot total\ discounted\ LY^U]$$

$$-\ [Cost^T(total\ discounted\ LY^T) - Cost^U(total\ discounted\ LY^U)]$$

[3] We implement a continuous time framework rather than a discrete time framework because the exact time horizon in the CEA studies being evaluated is not consistently reported (i.e., CEA studies commonly use a lifetime horizon, which could correspond to any number of years in practice; the exact number of years modeled is generally not reported). While we can replicate CEA results (total discounted QALYs and total discounted costs) using both discrete and continuous frameworks, we found that the latter yields more accurate estimates of intermediate inputs. Specifically, the continuous time framework implemented using an indefinite integral yields estimates of the total discounted LYs that are closer to the reported values in the CEA study, relative to a discrete time framework implemented using an infinite geometric series.

[4] Total undiscounted LYs are preferable to total discounted LYs for parameterizing the survival function because they avoid confounding the mortality hazard estimate with discounting. However, when undiscounted LYs are not reported in a CEA study, total discounted LYs provide a reasonable substitute for the implementation.

© 2025 Mulligan K, et al. *JAMA Health Forum.*

Using the fact that costs are the same for both GRACE and CEA, we can simplify the above expression to the following:

Equation 2.3 Simplified GRACE NMB

$$NMB_{GRACE} = \frac{K}{W(1-d^*)}\left[W\left(H_{sj}+B\right)\cdot total\ discounted\ LY^T - W(H_s)\cdot total\ discounted\ LY^U\right] - \ total\ incremental\ discounted\ cost$$

A key takeaway from this final simplification is that we only require information on total costs (i.e., we do not need period-level costs) from CEA to implement GRACE costs. Similarly, we can revise the GRACE decision rule in equation 2.2 to obtain the simplified form:

Equation 2.4 Simplified GRACE Decision Rule

$$K\frac{1}{W(1-d^*)} > \frac{total\ incremental\ discounted\ cost}{[W(H_s+B)(total\ discounted\ LY^T) - W(H_s)(total\ discounted\ LY^U)]}$$

2.2 Value-based Price (VBP) Derivation

If data on the total drug cost of the intervention drug are provided in the CEA report, we can derive VBPs under the simplifying assumptions used to derive equations 2.3 and 2.4. Let the VBP for the intervention drug (VBP^T) be defined as the drug price which sets $NMB_{GRACE} = 0$. To conserve notation, define:

$$B_{GRACE} = \frac{K}{W(1-d^*)}[W(H_s+B)\cdot total\ discounted\ LY^T - W(H_s)\cdot total\ discounted\ LY^U]$$

Then:

$$NMB_{GRACE} = B_{GRACE}\ -\ [total\ discounted\ cost^T - total\ discounted\ cost^U]$$

For the intervention arm, we can decompose the total discounted costs as

$$total\ discounted\ cost^T = (total\ discounted\ drug\ cost)^T + (total\ discounted\ nondrug\ cost)^T$$

Note that when $NMB_{GRACE} = 0$, then $total\ discounted\ drug\ cost^T \equiv VBP^T \cdot (total\ discounted\ LY^T)$. We can rearrange terms to obtain a simplified expression for VBP^T:

Equation 2.5 Simplified GRACE VBP

$$VBP^T = \left(\frac{1}{total\ discounted\ LY^T}\right)[B_{GRACE} + (total\ discounted\ cost^U) - (total\ discounted\ nondrug\ cost^T)]$$

3. Standard CEA Implementation Framework

Under standard CEA, we assume that total benefits correspond to total discounted QALYs. Using equation 2.3, we can obtain a comparable NMB expression under standard CEA if we assume a linear health utility function such that $W(H) = H$ and no disability adjustment (i.e., $d^* = 0$):

Equation 3.1 Standard CEA NMB

$$NMB_{CEA} = K\left[\left(H_{sj}+B_j\right)\cdot total\ discounted\ LY^T - (H_s)\cdot total\ discounted\ LY^U\right] - total\ incremental\ discounted\ costs$$

Similarly, the standard CEA decision rule can be expressed as follows:

Equation 3.2 Standard CEA Decision Rule

$$K > \frac{total\ incremental\ discounted\ costs}{[(H_s+B)(total\ discounted\ LY^T) - (H_s)(total\ discounted\ LY^U)]}$$

© 2025 Mulligan K, et al. *JAMA Health Forum*.

Define the standard CEA VBP (VBP^T_{CEA}) to be the drug price when $NMB_{CEA} = 0$. In cases where we can obtain data on the total drug cost associated with the intervention arm, we can obtain VBPs under the standard CEA approach as follows. To conserve notation, define:

$$B_{CEA} = K\left[\left(H_{sj} + B_j\right) \cdot total\ discounted\ LY^T - (H_s) \cdot total\ discounted\ LY^U\right]$$

Then:

$$NMB_{CEA} = B_{CEA} - \left[total\ discounted\ cost^T - total\ discounted\ cost^U\right]$$

Similar to the GRACE derivation of VBP, we can decompose the total costs in the intervention arm into the total drug cost and total non-drug costs. When $NMB_{CEA} = 0$, $total\ discounted\ drug\ cost^T \equiv VBP^T_{CEA} \cdot total\ discounted\ LY^T$. We can rearrange terms to obtain a simplified expression for VBP^T_{CEA}. Then:

Equation 3.3 Standard CEA VBP

$$VBP^T_{CEA} = \left(\frac{1}{total\ discounted\ LY^T}\right)\left[B_{CEA} + (total\ discounted\ cost^U) - (total\ discounted\ nondrug\ cost^T)\right]$$

4. Empirical Implementation

4.1. Data Requirements

In absence of de novo models, to obtain GRACE estimates for NMB (equation 2.3) and the GRACE decision rule (equation 2.4), we require the following information from an existing CEA study: total undiscounted LY in the intervention and comparator arms (or the total discounted LYs in the intervention and comparator arms), the discount rate, total discounted incremental cost (or total discounted cost in the intervention and comparator arms), and total discounted QALYs in the intervention and comparator arms.[5] VBP calculations also require information on the total discounted drug cost in the intervention arm. We can use these same inputs to replicate the published CEA results by implementing equations 3.1-3.3. Table 2 summarizes which pieces of CEA information are used to derive each GRACE input.

[5] We note that total discounted QALYs do not appear in equations 2.3-2.5 or the analogous CEA equations. However, we will use this information to construct estimates of health in the intervention and comparator arms ($(H_S + B)$ and H_S, respectively).

Table 2. GRACE Equation Inputs and Required CEA Information

Equation term(s) [relevant equation numbers]	Description	Required CEA study information
$total\ discounted\ LY^T$ and $total\ discounted\ LY^U$<br><br>[Equations 2.3-2.5; 3.1-3.3]	Total discounted LY in the intervention and comparator arms	• Total undiscounted LY in the intervention and comparator arms OR total discounted LY in the intervention and comparator arms<br>• Discount rate
$(H_s + B)$ and (H_s)<br><br>[Equations 2.3-2.5; 3.1-3.3]	Health in the intervention and comparator arms	• Total undiscounted LY in the intervention and comparator arms OR total discounted LY in the intervention and comparator arms<br>• Total discounted QALYs in the intervention and comparator arms<br>• Discount rate
$total\ incremental\ discounted\ costs$<br><br>[Equations 2.3, 2.4; 3.1, 3.2]	Difference between total discounted costs associated with the intervention and comparator arm	• Total discounted incremental cost OR total discounted cost in the intervention and comparator arms
$total\ \ discounted\ nondrug\ cost^T$<br><br>[Equations 2.5 and 3.5]	Total discounted non-drug cost in the intervention arm (used for VBP calculation)	• Total discounted drug cost in the intervention arm<br>• Total discounted cost in the intervention arm

4.2. Mapping Published CEA Information to GRACE Inputs

In this section we refer to information extracted from a CEA study as a "reported" value (e.g., reported total discounted LY in the comparator arm) to distinguish them from values used in the GRACE implementation equations (e.g., $total\ discounted\ LY^U$).

We first use the reported total undiscounted LY in the intervention and comparator arms to parameterize the exponential survival function parameter (λ^i) for *i* equal to T, U. Recall based on our implementation assumptions, we have

$$\lambda^i = \frac{1}{reported\ total\ undiscounted\ LY^i} \text{ for } i \text{ equal to T, U.}$$

If reported total undiscounted LY information is not available in the CEA study, we can instead derive the survival function parameter using reported total discounted LYs and the discount rate. Based on our implementation assumptions, we have

$$\lambda^i = \frac{1}{reported\ total\ discounted\ LY^i} - r \text{ for } i \text{ equal to T, U.}$$

We then use the estimate for λ^i along with the reported discount rate (r) to calculate $total\ discounted\ LY^i$ for *i equal to T, U* (corresponds to row 1 in Table 2):

$$total\ discounted\ LY^i = \frac{1}{\lambda^i + r} \text{ for } i \text{ equal to T, U.}$$ [6]

Now that we have estimates for $total\ discounted\ LY^T$ and $total\ discounted\ LY^U$, we can derive the health levels for the intervention and comparator arms (corresponding to row 2 in Table 2). In particular, we have:

[6] When we use reported total discounted LYs to derive the survival parameter, the calculated value for total discounted LYs is mechanically equivalent to the reported total discounted LYs. However, this will not always be the case if a discrete time framework is used.

© 2025 Mulligan K, et al. *JAMA Health Forum*.

$$H_s = \frac{reported\ total\ discounted\ QALY^U}{total\ discounted\ LY^U}$$

$$(H_s + B) = \frac{reported\ total\ discounted\ QALY^T}{total\ discounted\ LY^T}$$

The calculations to obtain total incremental discounted cost and total non-drug cost in the intervention arm (corresponding to rows 3 and 4 in Table 2) are trivial in the sense that we can simply use the reported total incremental discounted cost and reported total discounted non-drug cost from the CEA report.

In addition to the above inputs, we need estimates for the WTP threshold (K), the disability adjustment (d^*), and the utility function (W). Values and sources for these GRACE inputs are provided in Table 3. For this study, we rely on the power utility function because it has been shown to be non-discriminatory and is therefore compliant with the Inflation Reduction Act.[4] The results do not change much if we use a two-parameter expo power utility function and are available upon request from the authors.

Table 3. Additional GRACE Model Inputs

**Mathematical terms**	**Description**	**Empirical value (Source)**
K	WTP threshold from CEA	$150K [5] [7]
d^*	Health loss from disability	0.149 [2]
$W(H)$	Exact utility function over health	Power heath utility function: $W(H) \equiv \frac{H^{1-\rho}}{1-\rho}$ where: $\rho = 0.2822$ (Refer to Table 4 in Mulligan et al. [3])

[7] The 2023 ICER value assessment framework usually adopts a range of $100-$150k USD per QALY for WTP thresholds (Source: ICER 2023)

5. Example of GRACE and CEA Model Inputs and Outcomes

We do not report a full list of all inputs for our study because the specific model input values vary by CEA. To provide an example of the implementation, we present the relevant inputs and calculated GRACE outcomes for the published CEA comparing lumacaftor/ivacaftor (intervention) to best supportive care (BSC; comparator) for the treatment of cystic fibrosis.[6] The specific model inputs correspond to the subgroup analysis of the population homozygous for the F508del mutation (age 2). All model inputs for this example are summarized in Table 4.

We implement the simplified GRACE and standard CEA approximations in two steps. First, we compute all intermediate inputs shown in Table 4 following the mapping outlined in Section 4.2. Second, we compute the outcomes of interest: NMB, ICER, GRACE-ICER, and VBPs according to equations 2.3-2.5 (GRACE) and equations 3.1-3.3 (CEA). Outcomes for this example are reported in Table 5.

Table 4. GRACE and CEA Model Inputs (Lumacaftor/Ivacaftor Compared to BSC)

Input	Value		Source
Power utility function parameter (ρ)	0.2822		[3]
Disability adjustment (d^*)	0.149		[2]
Discount rate (r)	3%		[6]
Base currency year	2020		
K	$150,000		
	**Intervention:** Lumacaftor/Ivacaftor	**Comparator:** BSC	
Reported total undiscounted LY	49.94	37.05	
Reported total discounted LY	25.22	21.46	
Reported total discounted QALYs	19.43	15.77	
Reported total discounted costs	7,508,000	2,088,000	
Reported total discounted drug cost	6,212,000	Not required	
Survival function parameter (λ)	0.020	0.027	Calculated
Total discounted LY [8]	19.99	17.54	Calculated
Health (($H_s + B$) and H_s)	0.97	0.89	Calculated
Utility over health ($W(H)$)	0.98	0.92	Calculated

Notes: All costs are in USD. The currency year is used to inflate results to 2024 USD. In instances where ICER does not report the currency year, we assume it is equal to the year of the report's publication date. The method and formulae used to obtain calculated values is outlined in Section 4.2. Utility over health values are normalized to range from 0 to 1 as described in Mulligan et al. [3].

Table 5. GRACE and CEA Model Outcomes

	Equation	Value (2020 USD)	Inflation-adjusted value (2024 USD) [9]
**Standard CEA** [10]			
Net monetary benefit	2.1	-4,871,000	-5,903,834
Incremental CE ratio	2.2	1,480,874	1,794,875
Value-based price	2.3	67,082	81,306
**GRACE**			
Net monetary benefit	1.3	-4,858,463	5,888,639
Incremental CE ratio	1.4	1,202,532	1,457,514
Value-based price	1.5	67,709	82,066

[8] Note that calculated values of total discounted life-years may not exactly match the reported total discounted life-years (as shown in an earlier row of Table 4).

[9] All values were inflation-adjusted to USD 2024 values using the average annual CPI-U (Source: Bureau of Labor Statistics, 2024).

[10] The reported incremental CE ratio was equal to $1,480,000 (2020 USD), which is 0.06% lower than our estimated incremental CE ratio. The difference is likely the result of ICER rounding the results in their report to the nearest hundred thousand.

© 2025 Mulligan K, et al. *JAMA Health Forum*.

6. Additional Results

6.1. Scenario Analysis: Value of a QALY

Table 6. Spending Distribution by Disease Severity, All Drugs

Standard of care health level (0-100)	CEA [N=69]				GRACE [N=69]			
	K=$50,000	K=$100,000	K=$150,000	K=$200,000	K=$50,000	K=$100,000	K=$150,000	K=$200,000
<60 (N=12)	3.35%	3.64%	3.78%	3.87%	3.76%	4.19%	4.41%	4.55%
60-69 (N=19)	2.69%	2.40%	2.25%	2.16%	2.67%	2.39%	2.24%	2.15%
70-79 (N=17)	5.10%	5.72%	6.05%	6.25%	5.64%	6.46%	6.88%	7.14%
80-100 (N=21)	88.87%	88.24%	87.92%	87.72%	87.92%	86.96%	86.47%	86.17%
Total spend (USD, billions)	$231.8	$338.1	$444.5	$550.8	$234.8	$344.1	$453.4	$562.7

Notes: Analysis includes drugs with non-missing data for population size and VBP. We assume the drug price is equal to VBP and 100% of the eligible population receives treatment.

Table 7. Spending Distribution by Disease Severity, Cost-effective Drugs

Standard of care health level (0-100)	CEA [N=25]				GRACE [N=27]			
	K=$50,000	K=$100,000	K=$150,000	K=$200,000	K=$50,000	K=$100,000	K=$150,000	K=$200,000
<60	7.47%	4.38%	12.71%	11.32%	6.58%	3.74%	13.03%	12.59%
60-69	4.91%	3.78%	4.01%	5.07%	4.60%	3.57%	2.93%	4.60%
70-79	2.09%	2.97%	2.48%	2.24%	1.92%	2.04%	2.54%	2.32%
80-100	85.53%	88.87%	80.80%	81.37%	86.91%	90.65%	81.51%	80.49%
Total spend (USD, billions)	$36.6	$65.7	$103.6	$144.5	$40.0	$71.4	$120.7	$162.5

Notes: Analysis includes drugs with non-missing data for population size and VBP. We assume the drug price is equal to VBP and 100% of the eligible population receives treatment.

© 2025 Mulligan K, et al. *JAMA Health Forum*.

Table 8. Spending Distribution by Disease Severity, Cost-ineffective Drugs

Standard of care health level (0-100)	CEA [N=44]				GRACE [N=42]			
	K=$50,000	K=$100,000	K=$150,000	K=$200,000	K=$50,000	K=$100,000	K=$150,000	K=$200,000
<60	2.58%	3.46%	1.07%	1.23%	3.18%	4.31%	1.28%	1.28%
60-69	2.27%	2.07%	1.72%	1.12%	2.28%	2.08%	1.99%	1.15%
70-79	5.66%	6.39%	7.13%	7.68%	6.41%	7.61%	8.46%	9.10%
80-100	89.49%	88.09%	90.08%	89.97%	88.13%	86.00%	88.27%	88.47%
Total spend (USD, billions)	$195.2	$272.5	$340.9	$406.4	$194.8	$272.7	$332.7	$400.1

Notes: Analysis includes drugs with non-missing data for population size and VBP. We assume the drug price is equal to VBP and 100% of the eligible population receives treatment.

6.2. Robustness Check: Uniform Average Health Assumption

The main analysis assumes a uniform health each period (Q). We estimated sensitivities that assume health gains in each arm (intervention and comparator) are front loaded. To do this, we split the lifetime horizon into two parts with durations L1 and L2. L1 has an average health equal to $Q1 = Q * M$, where Q is the baseline health from the main scenario, and M is a multiplier > 1. We solve for Q2 given values of L1, L2, Q, and total lifetime QALYs. We considered scenarios where L1 equaled 1 and 10, and M equaled 1.05, 1.10, and 1.20. For L1 = 10, Q2 is negative for some drugs (N=18 when M=1.05 and N=29 when M=1.2). This results in a missing value for GRACE VBP because utility cannot be calculated for negative health levels. The budget estimates for these scenarios exclude the drugs with missing GRACE VBPs.

Table 9. Sensitivity Analysis: Uniform Health Assumption Budget Impact

	Main analysis	(1)	(2)	(3)	(4)	(5)
Total budget CEA ($, B)	444.48	444.48	444.48	444.48	442.05	434.11
Total budget GRACE ($, B)	453.38	453.38	453.39	453.53	446.22	445.79
Difference ($, B)	8.90	8.90	8.91	9.05	8.77	11.68
% difference	2.0%	2.0%	2.0%	2.0%	2.0%	2.7%
Time horizon for front-loading (L1)	N/A	1	1	1	10	10
Multiplier for Q1	N/A	1.05	1.100	1.200	1.05	1.20

Notes: Scenarios 4 and 5 exclude the N=18 and N=29 drugs with missing values for GRACE VBPs.

Figure 1. Sensitivity Analysis: Uniform Health Assumption Percentage difference in GRACE and CEA VBP

Notes: Left panel shows results for uniformly distributed QoL and three scenarios with a time horizon for front-loading (L1) equal to 1 and includes 185 observations with VBPs. Right panel shows results for uniformly distributed QoL (N=185), and three scenarios with a time horizon for front-loading (L1) equal to 10 (with non-missing VBPs for N=150, 144, and 131 for multipliers equal to 1.05, 1.1, and 1.2, respectively).

© 2025 Mulligan K, et al. *JAMA Health Forum*.

Figure 2. Sensitivity Analysis: Uniform Health Assumption Percentage Difference in GRACE and CEA VBP (Population Weighted)

Notes: Left panel shows population-weighted results for uniformly distributed QoL and three scenarios with a time horizon for front-loading (L1) equal to 1 and includes 84 observations with VBPs and population sizes. Right panel shows population-weighted results for uniformly distributed QoL (N=84), and three scenarios with a time horizon for front-loading (L1) equal to 10 (with non-missing VBPs for N=64, 58, and 52 for multipliers equal to 1.05, 1.1, and 1.2, respectively).

© 2025 Mulligan K, et al. *JAMA Health Forum*.

References

1. Lakdawalla DN, Phelps CE. The generalized risk-adjusted cost-effectiveness (GRACE) model for measuring the value of gains in health: an exact formulation. Journal of Benefit-Cost Analysis. 2023;14(1):44–67.

2. Pickard AS, Law EH, Jiang R, Pullenayegum E, Shaw JW, Xie F, et al. United States valuation of EQ-5D-5L health states using an international protocol. Value in health. 2019;22(8):931–41.

3. Mulligan K, Baid D, Doctor JN, Phelps CE, Lakdawalla DN. Risk preferences over health: empirical estimates and implications for medical decision-making. Journal of Health Economics. 2024;94:102857.

4. Lakdawalla DN, Doctor JN. A principled approach to non-discrimination in cost-effectiveness. Eur J Health Econ. 2024 Nov;25(8):1393–416.

5. Vanness DJ, Lomas J, Ahn H. A Health Opportunity Cost Threshold for Cost-Effectiveness Analysis in the United States. Ann Intern Med. 2021 Jan;174(1):25–32.

6. Institute for Clinical and Economic Review. Modulator Treatments for Cystic Fibrosis: Effectiveness and Value [Internet]. 2020 Sep. Available from: https://icer.org/assessment/cystic-fibrosis-2020/

© 2025 Mulligan K, et al. *JAMA Health Forum*.
