# Supplementary material for: Measuring the Budget Impact of Nondiscriminatory Cost-Effectiveness
Source: JAMA Health Forum. 2025 Sep 5;6(9):e253076. doi: 10.1001/jamahealthforum.2025.3076 (PMC12413641; doi:10.1001/jamahealthforum.2025.3076)
Supplement: Supplement 2. — Data Sharing Statement [file jamahealthforum-e253076-s002.pdf]

Data Sharing Statement

Mulligan. Measuring the Budget Impact of Nondiscriminatory Cost-Effectiveness. *JAMA Health Forum*. Published September 05, 2025. doi:10.1001/jamahealthforum.2025.3076

Data
Data available: No

Additional Information
Explanation for why data not available: The data used in our study is publicly available in reports published on the Institute for Clinical and Economic Review website.
